# Galectin-3 disruption impaired tumoral angiogenesis by reducing VEGF secretion from TGF*β*1-induced macrophages

**DOI:** 10.1002/cam4.173

**Published:** 2014-01-12

**Authors:** Camila Maria Longo Machado, Luciana Nogueira Sousa Andrade, Verônica Rodrigues Teixeira, Fabrício Falconi Costa, Camila Morais Melo, Sofia Nascimento dos Santos, Suely Nonogaki, Fu-Tong Liu, Emerson Soares Bernardes, Anamaria Aranha Camargo, Roger Chammas

**Affiliations:** 1 Laboratório de Oncologia Experimental—LIM24, Departamento de Radiologia e Oncologia, Faculdade de Medicina, Universidade de São PauloSão Paulo, Brazil; 2 Depto. de Radiologia e Oncologia, Centro de Investigação Translacional em Oncologia, Instituto do Câncer do Estado de São Paulo, Faculdade de Medicina, Universidade de São PauloSão Paulo, Brazil; 3 Laboratório de Investigação Médica Radioisotopos—LIM/43, Departamento de Radiologia e Oncologia, Faculdade de Medicina, Universidade de São PauloSão Paulo, Brazil; 4 Cancer Biology and Epigenomics Program, Ann & Robert H. Lurie Children's Hospital of Chicago Research Center and Department of Pediatrics, Northwestern University's Feinberg School of MedicineChicago, IL, 60614; 5 Departamento de Patologia, Instituto Adolfo LutzSão Paulo, Brazil; 6 Institute of Biomedical Sciences, Academia SinicaTaipei, Taiwan; 7 Instituto Ludwig de Pesquisa sobre o CâncerSão Paulo, Brazil

**Keywords:** Angiogenesis, galectin-3, melanoma, tumor microenvironment

## Abstract

In order to study the role of galectin-3 in tumor angiogenesis associated with tumor-associated macrophages (TAM) and tumor parenchyma, the galectin-3 expression was reconstituted in Tm1 melanoma cell line that lacks this protein. Galectin-3-expressing cells (Tm1G3) and mock-vector transfected cells (Tm1N3) were injected into wild-type (WT) and galectin-3 knockout (KO) C57Bl/6 mice. Tumors originated from Tm1G3 were larger in tumor volume with enlarged functional vessels, decreased necrotic areas, and increased vascular endothelial growth factor (VEGF) protein levels. Galectin-3-nonexpressing-cells injected into WT and KO showed increased levels of transforming growth factor beta 1 (TGF*β*1) and, in WT animals this feature was also accompanied by increased VEGFR2 expression and its phosphorylation. In KO animals, tumors derived from galectin-3-expressing cells were infiltrated by CD68^+^-cells, whereas in tumors derived from galectin-3-nonexpressing-cells, CD68^+^ cells failed to infiltrate tumors and accumulated in the periphery of the tumor mass. In vitro studies showed that Tm1G3 secreted more VEGF than Tm1N3 cells. In the latter case, TGF*β*1 induced VEGF production. Basal secretion of VEGF was higher in WT-bone marrow-derived macrophages (BMDM) than in KO-BMDM. TGF*β*1 induced secretion of VEGF only in WT-BMDM. Tm1G3-induced tumors had the Arginase I mRNA increased, which upregulated alternative macrophage (M2)/TAM induction. M2 *stimuli*, such as interleukin-4 (IL4) and TGF*β*1, increased Arginase I protein levels and galectin-3 expression in WT- BMDM, but not in cells from KO mice. Hence, we report that galectin-3 disruption in tumor stroma and parenchyma decreases angiogenesis through interfering with the responses of macrophages to the interdependent VEGF and TGF*β*1 signaling pathways.

## Introduction

Galectin-3, a conserved *β*-galactoside-binding animal lectin, has important physiological roles, such as development and activation of the immune system, besides its involvement in tumor pathophysiology 1. Tumor-associated angiogenesis is a critical and targetable step of tumorigenesis, resulting from the action of different cytokines and growth factors (e.g., VEGF 2) produced by a variety of cellular elements within the tumor microenvironment. Different groups have suggested that galectin-3 plays a role in controlling tumor-associated angiogenesis.

In experiments with Human Umbilical Vein Endothelial Cells (HUV-EC-C), Nangia-Makker et al. 3 described that neutralizing galectin-3 by specific carbohydrates and anti-galectin-3 antibodies affect chemotaxis, endothelial cell morphology, and capillary tube formation in vitro. These authors also demonstrated that galectin-3 has pro-angiogenic activity, which may be related to its ability to induce endothelial cell migration. In addition, they showed that xenotransplantation of galectin-3-overexpressing human breast ductal carcinoma cells in nude mice promotes tumor angiogenesis more efficiently. Markowska et al. 4 showed in vitro that galectin-3 siRNA knockdown as well as galectin-3 blockers resulted in reduction in angiogenesis induced by VEGF and basic fibroblast growth factor (bFGF). These authors demonstrated that VEGF and bFGF-induced angiogenesis in mouse corneal micropocket assay was reduced in galectin-3 knockout (KO) mice. They provided evidence to support the notion that galectin-3 generates VEGFR2-clusters, thus potentiating cell signaling effects in endothelial cells 5.

Release of VEGF within the tumor microenvironment recruits bone marrow-derived cells, which turn into tumor-associated macrophages (TAM), further enhancing the local production of VEGF 6. The amount of TAM has been associated with poor prognosis and outcome by increased macrophage gene transcription levels (e.g., CD68) in breast carcinomas and lymphomas 7,8. In general, TAMs display an M2 phenotype, which has pro-tumoral functions, such as promoting stromal modifications allowing for tumor cell survival, proliferation, and spread. In in vitro studies, MacKinnon et al. 9 demonstrated that galectin-3 is necessary for optimal M2 activation. On the other hand, Dragomir et al. 10 demonstrated that toxic stimuli in vivo overrides galectin-3 deficiency in hepatic macrophages from galectin-3-disrupted mice, as these cells could be induced to alternative activation. The question of how and to what extent galectin-3 contributes to M2 polarization thus remains open.

VEGF is also augmented by mediators produced by TAM and tumor cells 11 such as transforming growth factor beta 1 (TGF*β*1) and other factors, such as IL-13, IL-4, and IL-10 6, skewing the macrophage response toward an M2 phenotype. In high concentrations, TGF*β*1 may inhibit endothelial cell growth in vitro 12 and in vivo 2. Furthermore, TGF-*β*1 induces angiogenesis by stimulating the production of positive regulators from stromal cells and through chemoattraction of bone-marrow-derived monocytes 2. TGF*β*1 is strongly induced in hypoxic tissues 13 and its induction results in increased tumor progression and peritumoral angiogenesis in melanomas 14. TGF*β*1 also attracts macrophages directly as a potent chemoattractant 15 or indirectly by inducing CCL5/regulated on activation, normal T cell expressed and secreted 16 production by stromal cells.

Our group has established a tumorigenic melanoma model (Tm1 cells) derived from a non-tumorigenic cell line named Melan-A 17,18. Transcriptome analysis showed that Tm1 cells lost galectin-3 expression 19. Here, we have analyzed the impact of heterologous xenogenic expression of galectin-3 in Tm1 cells engrafted in both wild-type and galectin-3 null mice to address the role of galectin-3, and its cellular origin, in melanoma tumor growth and tumor-associated angiogenesis.

## Material and Methods

### Cell culture

All cell lines were grown in RPMI-1640, pH-6.9 supplemented with 5% heat-inactivated fetal calf serum (Gibco, Life Technologies, Carlsbad, CA), as described elsewhere 17,18. Tm1 cells were transfected with pEF1neo plasmidial vector containing human cDNA for galectin-3 or not, originating the cell lines Tm1G3 (galectin-3-positive) and Tm1N3 (galectin-3-negative). All transfections were performed using lipofectamine (Gibco, Life Technologies) according to the manufacturer's protocol. Transfected cells were maintained in RPMI 1640 containing 5% of fetal bovine serum and 1 mg/mL geneticin (G418; Sigma-Aldrich, St. Louis, MO). Expression of galectin-3 in each cell line/clone was determined by immunoblotting as detailed in the supporting information.

### DNA methylation analysis

Genomic DNA from melan-A and Tm1 cells were obtained by digestion with proteinase K (100 *μ*g/mL; Life Technologies) and RNAse (20 *μ*g/mL; Life Technologies) for 16 h at 50°C, followed by phenol/chloroform/isoamyl alcohol extraction. The presence of CpG islands in galectin-3 promoter sequence was detected by the bisulfite genome sequencing method, essentially as described by others 20 and as given in supporting information.

### Animals and tumoral growth evaluation

All procedures were in accordance with ethical principles adopted by the Brazilian College of Animal Experimentation and approved by the Ethical Committee for Animal Research of School of Medicine, University of São Paulo (#089/09). Eight-week-old male C57black/6 wild-type (WT) or galectin-3 KO mice 21 received a subcutaneous inoculation of 2 × 10^5^ cells in right flank and tumor growth was determined as described before 22. After euthanasia, tumors were collected for routine histopathology, immunohistochemistry, and immunofluorescence followed by quantification as described in the supporting information. A section measuring 1 mm from the tail was collected for galectin-3 genotyping as described by Doverhag et al. 23.

### Western blotting

Total protein extracts were eletrophoretically separated in sodium dodecyl sulfate polyacrylamide gel electrophoresis (SDS-PAGE) and blotted onto polyvinylidene difluoride (Hybond-P; GE Healthcare, Little Chalfont, U.K.), membrane according to standard procedures as described by others 24–26. The antibodies used were: anti-galectin-3 (M3/38 Hybridoma), anti-VEGF (1:500; Santa Cruz Biotechnology, Santa Cruz, CA), anti-VEGFR2, and its anti-phosphorylated form, pY1214-VEGFR2 (1:500; Invitrogen, Life Technologies, Carlsbad, CA), TGF*β*1 (1:3000; BD Biosciences—Pharmingen in San Diego, CA) or Arginase I (1:1000; BD Biosciences—Pharmingen); followed by specific HRP (horseradish peroxidase)-labeled secondary antibody (Sigma-Aldrich, 1:4000). The procedures are detailed in the supporting information section.

### Total RNA extraction, reverse transcription, and real-time qRT-PCR

Tumors from at least three individuals were sectioned into four quadrants and total RNA was extracted from tissue sections using Tryzol reagent (Invitrogen, Life Technologies). The RNA was purified with RNeasy mini-kit (Qiagen, Austin, TX) according to the manufacturer's protocol. Reverse transcription and quantitative polymerase chain reaction (qPCR) were performed using 10 ng of RNA from each sample using SuperScript® III One-Step RT-PCR System (Invitrogen, Life Technologies) following manufacturer's instructions published elsewhere. qPCR was carried out in a Rotor gene 6000 detection system equipped with a SYBR Green fluorescence detector for amplicon quantification. The target primers used for qPCR are presented in Table S1. The relative expressions, of each gene were obtained by GeNorm^plus^ algorithm from Vandesompele et al. 27 comparing target genes to endogenous controls (*β*-actin, *β*II-microglobulin, TATA binding protein, RpLP3a, and RpLP0).

### Macrophage cultures and in vitro experiments

Total cells were extracted from femoral or tibial bone marrow from both WT and KO mice. These cells were cultivated in RPMI 1640 media (Gibco, Life Technologies) supplemented with 20% heat-inactivated fetal calf serum (Gibco, Life Technologies), 1% of penicillin and streptomycin (Sigma-Aldrich) and 30% of L-Cell Conditioned Media as a source of Macrophage Colony Stimulating Factor (M-CSF) for 7–9 days. After this period, the medium was removed and changed to RPMI 1640 media (Gibco, Life Technologies) supplemented with 20% heat-inactivated fetal calf serum (Gibco, Life Technologies), 1% of penicillin and streptomycin (Sigma-Aldrich) with specific M1 (Lipopolysaccharide [LPS] 1 *μ*g/mL (Sigma-Aldrich) and IFN-*γ* 50 ng/mL; (R&D System, Minneapolis, MN) or M2 (IL-4 50 ng/mL (R&D System)or TGF-*β*1 50 ng/mL, (R&D System) stimuli. To identify BMDMs (bone marrow-derived macrophage) cells were incubated with *Fc-*block (conditioned media) followed by murine—anti-F4/80 (macrophage pan marker)—PE incubation (Caltag Laboratories South San Francisco, CA) and then visualized by flow cytometry in a FACs-Calibur (BD Biosciences—Pharmingen). These experiments showed that these adherent cells were >93% of F4/80 positive in WT and >95% of KO cells in each 10^7^ isolated cells.

### VEGF assay in BMDM or Tm1N3 and Tm1G3 conditioned media

VEGF quantification in conditioned media from BMDM (from both WT and KO mice) and Tm1N3 or Tm1G3 cells after stimuli were evaluated with an enzyme-linked immunosorbent assay (ELISA) method. Equal amounts of protein were measured using a mouse VEGF ELISA kit (Peprotech, Rocky Hill, NJ) that recognizes VEGF. VEGF recombinant concentrations were determined using a standard curve prepared with each experiment.

### Statistical analysis

For each experiment in vivo, the numbers of individuals were indicated as “*n*” in each figure legend. The in vitro experiments were carried out at least three/four times (in triplicate or quadruplicate), yielding similar results in each occasion. The most representative from all experiments conducted was chosen to compose the figures. Data were analyzed by unpaired *t*-test, one-way analyses of variance (ANOVA) or two-way ANOVA followed by post test (Bonferroni) and the differences were considered significant for *P* < 0.1(*), *P* < 0.01(**) or *P* < 0.001(***). All results are expressed as means ± SEM (standard error of the mean). All analyses were conducted using GraphPad Prism version 4.0 for Windows® (GraphPad® Software, San Diego, CA) and Microsoft Office® Excel software 2007 (Microsoft, Redmond, WA).

## Results

### Loss of galectin-3 was associated with its promoter methylation in Tm1 cells

**Figure 1:**
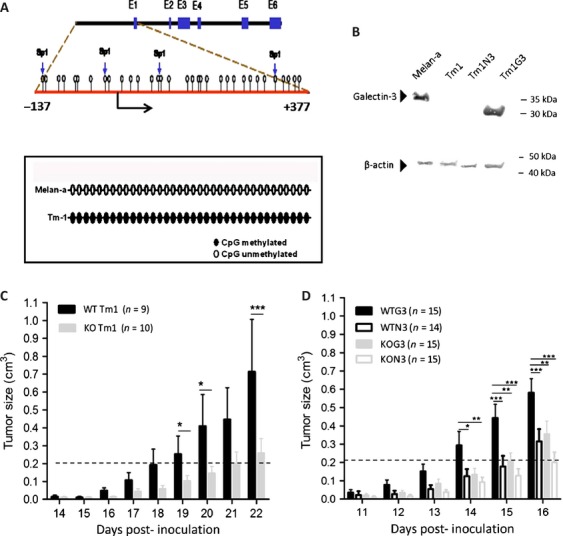
Methylation status of galectin-3 promoter region in melan-A and Tm1 cells. (A) Sequence of promoter, first exon and first intron (from −137 upstream to +377 downstream) of the galectin-3 gene (GenBank, # L08649). The analysis of these regions using CpGplot indicated that these regions lie within a putative CpG island. CpG dinucleotides methylated are represented as black circles whereas unmethylated CpG dinucleotides are represented in open circles. (B) Western blotting to galectin-3 in melan-A, Tm1 and transfected cells with a plasmid containing human cDNA for galectin-3 or the empty vector (Tm1G3 and Tm1N3) showing the presence of the murine galectin-3 in melan-A (molecular weight, ∼30 kDa), and the human galectin-3 in Tm1G3 (molecular weight, ∼25 kDa). Each lane corresponded to 20 *μ*g from total protein cell extracts. (C) Representative graph of tumor volume after Tm1-galectin-3-nonexpressing cells implantation in C57black/6 Lgals3^+/+^ (WT) or Lgal3s3^−/−^ (KO) mice. After 14 days post inoculation a palpable mass was detected. (D) After Tm1G3 injections in WT mice (WTG3) or KO mice (KOG3) a palpable mass was detected after 11 days post inoculation. The dashed line represents the cutoff tumoral volume to compare inocula of Tm1 and its transfected cells. The results in each graph corresponded to mean ± SEM and test used was two-way ANOVA with post test comparing all pair of columns. WT, wild type; KO, knockout; ANOVA, analyses of variance.

We evaluated the protein levels of galectin-3 in Tm1 cells, as compared to Melan-A and observed a total absence of this lectin in Tm1 cells, suggesting that galectin-3 expression was downregulated upon malignant transformation. It is well known that aberrant methylation of CpG dinucleotides is responsible for gene silencing 28 and, based on that, we first performed in silico analyses to identify putative CpG islands in the promoter region of the galectin-3 murine gene (GenBank sequence number L08649.1). Analysis of these sequences using CpG plot (EMBL) showed 33 CpG dinucleotides in the 5′ upstream region of the galectin-3 gene, including the first exon and first intron (Fig. 1A). The high content of CpG dinucleotides around regulatory regions of the galectin-3 gene suggests a possible role for DNA methylation in its control. To evaluate DNA methylation status of melan-A and Tm1 cell lines, we performed bisulfite genomic sequencing of a genomic region comprising the 33 CpG dinucleotides present in the 5′ upstream region of the galectin-3 gene. Sequencing of bisulfite-converted DNA revealed that all CpG dinucleotides were indeed methylated in the tumorigenic cell line and unmethylated in melan-A (Fig. 1A). Moreover, these regions had three extra CpG dinucleotides as compared to the actual galectin-3 sequence of Sv129 mice deposited in the GenBank.

We investigated the relationship between galectin-3 gene hypermethylation and its expression in vitro. The parental Tm1 cell line was treated with increasing concentrations (2.5, 5 or 10 *μ*mol/L) of 5′-Aza-2-deoxycytidine (5′-Aza-dCR, an inhibitor of DNA methyltransferases) for 72 h and we observed that galectin-3 expression was restored only with the highest concentration of 5′-Aza-dCR (Fig. S1A, B and C). Besides, a marked decrease in the original methylation pattern of the 5′ upstream region of the galectin-3 gene was observed after treatment of Tm1 cells with 10 *μ*mol/L of 5′-Aza-dCR (Fig. S1B). It is noteworthy that some CpG dinucleotides within putative SP1-binding sites become completely unmethylated after treatment (Fig. S1D). In addition, Western blot analysis showed that the levels of galectin-3 protein were partially restored after the treatment of Tm1 cells with 5′-Aza-dCR (Fig. S1D).

In order to access the impact of galectin-3 expression in melanoma engraftment and tumor growth, Tm1 cells were successfully transfected with a plasmid containing human cDNA for galectin-3 or the empty vector, generating stable clones, designated as Tm1G3 and Tm1N3, respectively. Galectin-3 expression levels were consistently checked by Western blotting throughout all the experiments, positive controls include analysis of murine galectin-3, expressed by melan-A cells (apparent molecular weight, ∼30 kDa) while in Tm1G3, expressing the human galectin-3 (∼25 kDa) (Fig. 1B), as described elsewhere 29. No functional differences are described between murine and human galectin-3. Galectin-3 was present in Tm1G3, whereas it was absent in the control transfectants (Tm1N3). Other stable clones were also selected and rendered essentially the same results shown for Tm1G3 and Tm1N3.

### Galectin-3 accelerates melanoma growth

The evaluation of how the lack of galectin-3 in melanoma cells impairs tumor growth was done by injecting Tm1 cells subcutaneously into both WT and galectin-3 KO C57Bl/6 mice. In fact, the absence of this lectin in both tumor cells and within the tumor microenvironment does not seem to interfere with the tumorigenic process as all galectin-3 KO mice injected with Tm1 cells developed tumors (Fig. 1C). However, tumor growth was significantly delayed in galectin-3 deficient mice.

We next injected the clones Tm1G3 or Tm1N3 into WT or KO mice to address whether the source of galectin-3 (tumor vs stromal origin) would impact on tumor engraftment and growth. Tm1G3 cells injected into WT mice (WTG3) grew faster in comparison to tumors originated from Tm1 parental cells or Tm1N3. Moreover, when the galectin-3-expressing cell line (Tm1G3) was injected into KO animals, a delay in tumor growth was also observed comparing with its growth in WT animals. Altogether, when galectin-3 was present in both tumor compartments and stromal compartments, tumors grew faster than any other combinations. On the other hand, when galectin-3 was absent in either tumor or stromal compartment, a significant reduction in tumor growth was observed (Fig. 1D).

### Presence of galectin-3 in both tumor cell and stromal cell compartments favors tumor-associated angiogenesis and balanced tumor growth

**Figure 2:**
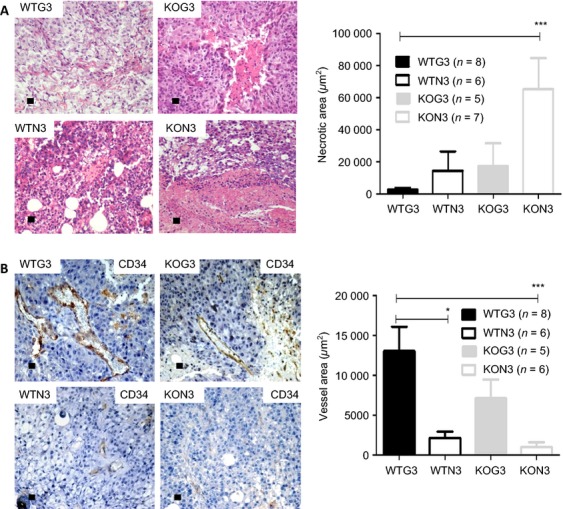
Morphological differences evaluation by Hematoxylin and Eosin stain and Immunohystochemistry. (A) Analysis of necrotic areas by H/E and (B) vascular density by counting CD34-positive cells per area. The results were submitted to unpaired *t-*test with *P < 0.1 and ***P < 0.001. The “*n*” value was indicated in the figure and each bar represents the mean ± SE. Graph from a representative experiment.

With the purpose of understanding differences in growth kinetic among all groups, necrotic areas as well as tumor vessels were evaluated by routine histopathology and using immunohistochemistry with anti-CD34 antibodies. We observed that tumors derived from G3 cells in WT or KO animals (WTG3 and KOG3, respectively) had smaller necrotic areas (Fig. 2A) and larger functional vessel areas (Fig. 2B). On the other hand, Tm1N3 tumors in KO mice (KON3) had larger intra-tumoral necrosis areas, which were accompanied by smallest functional vessel areas. Decrease in the relative area of functional vessels was also seen in Tm1N3 tumors in WT mice.

### Galectin-3 modifies VEGF expression elicited by TGF*β*1

To gain a mechanistic insight into the molecular mechanisms involved in the differences mentioned above, mRNA and proteins were collected from all groups after 16 days post inoculation to evaluate VEGF and TGF*β*1 expression levels within tumor microenvironment. Furthermore, mRNA of all above-mentioned conditions was analyzed using a dedicated microchip to evaluate the transcriptional profile of glycosylation-related genes. Regarding the molecular signatures of all the experimental conditions, as shown in supplementary data 2, the expression profile of the latter genes did not alter significantly, despite the differences observed in tumor growth, necrosis, and angiogenesis.

**Figure 3:**
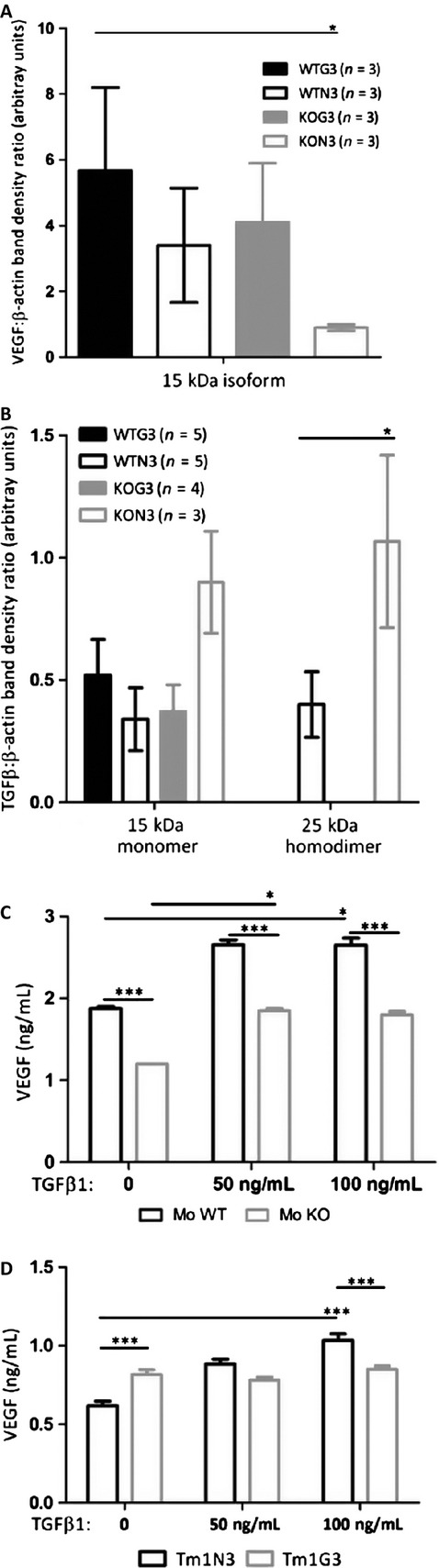
(A and B) VEGF and TGF*β*1 pro-angiogenic factors accumulation were analyzed by immunoblotting, followed by densitometric analyses of the blots using ImageJ. Each bar represents the mean ± SE of the values obtained (*n* represented within the figure). (C and D) Conditioned media from bone marrow-derived macrophages from WT or KO cells and Tm1N3 mock-cells or Tm1G3 galectin-3 transfected cells after 24 h of culture, dosed by ELISA of secreted VEGF to media (wells)/experiment (*n* = 6) in basal line (0) and after TGF*β*1 stimuli (50 ng/mL, 100 ng/mL). The results were submitted to unpaired test, two-tailed with *P < 0.1; ** *P* < 0.01 and ***P < 0.001. Each bar represents the mean ± SE and the graph is a representative result from four independent experiments. WT, wild type; KO, knockout; ELISA, enzyme-linked immunosorbent assay.

We further focused on the phenotypic differences observed and evaluated the protein levels of angiogenic mediators, such as VEGF and TGF*β*1. Corroborating our observations on the areas covered by functional vessels, secretion of VEGF was augmented in tumors with galectin-3 from either tumor or stromal origin. Tumors expressing galectin-3 in both stroma and parenchyma (WTG3) were the largest and contained the highest levels of detectable VEGF (Fig. 3A). In tumors without galectin-3 (KON3), we detected the smallest amount VEGF proteins (Fig. 3A). The expression of VEGF receptor, VEGFR2, was also analyzed and a higher amount of this receptor and its phosphorylated form was found in WT tumors when compared to tumors from KO mice. It is interesting to note that the smallest tumors, derived from galectin-3 nonexpressing cells growing in galectin-3 null environments (KON3), also showed the smallest amounts of VEGFR2 and its phosphorylated form (Fig. S3).

Based on the fact that TGF*β*1 improves peritumoral angiogenesis in melanomas 14 and acts indirectly as a potent chemoattractant for monocytes/macrophages 15, which releases VEGF in response of TGF*β*1 30, we investigated TGF*β*1 protein levels in tumors. While the unusual isoform of TGF*β*1 (∼15 kDa) was detected in all groups without any significant differences, the active TGF*β*1 homodimer (∼25 kDa) levels were increased in WTN3 and KON3 tumors. In order to test the hypothesis that disruption of galectin-3 in either macrophages or tumor cells could affect VEGF secretion in response to TGF*β*1 levels, we tested in vitro whether BMDM and/or cell lines secrete VEGF when cultured in TGF*β*1-enriched medium.

Basal VEGF secretion from WT-BMDM was higher than from KO-BMDM. Upon TGF*β*1 stimulation, a significant increase in VEGF secretion was observed (Fig. 3C) in WT-BMDM. Galectin-3 positive cells were consistently more responsive to TGF*β*1 than KO cells. Furthermore, TGF*β*1 led to accumulation of galectin-3 in WT-BMDM in a dose-dependent manner (Fig. S4A).

Regarding Tm1 clones, there was an increase in TGF*β*1-mediated VEGF secretion only by Tm1N3 cells, although the basal levels of secreted VEGF by Tm1G3 cells were higher (Fig. 3D). It is worth mentioning that TGF*β*1 increases did not cause any increase in galectin-3 levels in either cell clone, as a matter of fact galectin-3 in Tm1G3 decreased after TGF*β*1 stimuli (Fig. S4B).

### Galectin-3 disruption in stroma did not interfere with M2 enhancing signals in vivo

**Figure 4:**
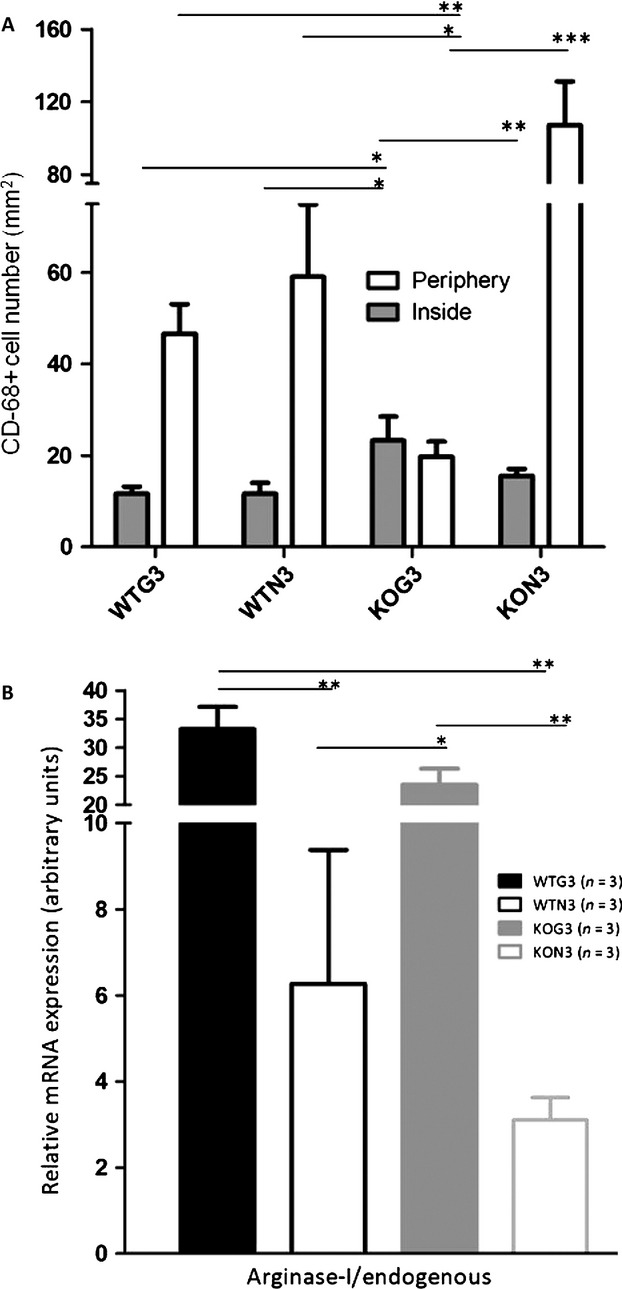
(A) Quantification of CD68-positive cells inside or in the periphery of WTG3, WTN3, KOG3, and KON3 tumors. Data were analyzed by one-way-ANOVA with *P < 0.1 and **P < 0.01. Each bar represents the mean ± SE (*n* = 3 for each experiment). (B) qPCR showing a M2-proned microenvironment by increased Arginase I mRNA in WTG3 and KOG3 tumors. Each bar represents the mean ± SE from three individuals, mRNA relative expression for each group, analyzed by t unpaired test, two-tailed with *P < 0.1; **P < 0.01 and ***P < 0.001. WT, wild type; KO, knockout; ANOVA, analyses of variance.

We performed immunofluorescence staining of macrophages associated with tumors was performed in cryopreserved tumoral tissues to determine the amount of CD68^+^ cells inside or in the periphery of tumor slices (as represented in Figs. 4A and S5). The results showed more infiltrating CD68^+^ cells in KOG3 tumors. On the other hand, in tumors derived from galectin-3 negative cells grew in galectin-3 deficient microenvironment (KON3 tumors), more peripheral CD68^+^ cells were observed.

As galectin-3 was shown before 9 as an important inducer of the M2 phenotype, we asked whether this lectin could modulate the phenotype of TAM within the tumor microenvironment. We addressed this point by measuring the mRNA levels of some mediators of both M2 (Arginase I, IL4 and IL10) and M1 (IL12p40, interferon gamma [INF*γ*]) phenotypes in tumors of all groups by qPCR. A significant difference was found in Arginase I mRNA levels. Indeed, we observed a fivefold increase in its level in the group with the largest tumors, WTG3 as compared to WTN3 tumors. It is interesting to note that in KOG3 tumors Arginase I mRNA levels had little difference from WTG3 (Fig. 4B). Regarding IL4, IL10, IL12-p40 and INF*γ* mRNA expression levels, no significant differences between WTG3, WTN3, KOG3 or KON3 were found (Fig. S6).

### BMDM from galectin-3 KO animals expressed higher levels of Arginase 1 but were insensitive to its modulation by M2 prototypical cytokines

Based on the increased expression of Arginase I in WTG3 tumors, as well as on the notion that tumor-associated macrophages are polarized to the protumorigenic M2 phenotype, we next tested the impact of galectin-3 disruption in this phenomenon. Once galectin-3 is regarded as a key molecule in this polarizing event 9, we studied the behavior of BMDM from both WT and KO mice after in vitro stimulation with IL-4 (50 ng/mL) or TGF*β*1 (50 ng/mL) pro-M2 stimuli and LPS 1 *μ*g/mL plus IFN-*γ* 50 ng/mL pro-M1 stimuli with or without addition of exogenous galectin-3 (50 *μ*g/mL).

**Figure 5:**
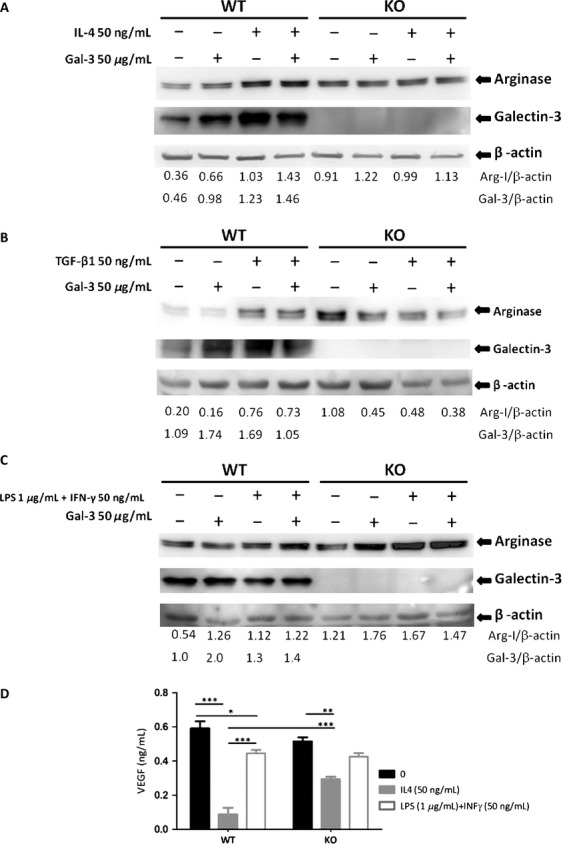
(A, B and C) Western blotting WT-BMDM or KO-BMDM of total protein cell extracts without stimulation or after IL-4 (50 ng/mL), TGF*β*1 (50 ng/mL) and LPS (1 *μ*g/mL) + IFN-*γ* (50 ng/mL), with or without exogen galectin-3 (50 *μ*g/mL). Each lane represents a pool from three independent assays (50 *μ*g/lane), each one performed with cells derived from one animal. The images were representative of two independent experiments. The number above each lane represents the target/*β*-actin relation from densitometric analysis performed using ImageJ. (D) The ELISA evaluated VEGF secreted in medium from bone marrow-derived macrophages WT or KO cells after 24 h of culture. The experiments were conducted in triplicates comparing basal levels with M2 polarization (IL-4, 50 ng/mL) or M1 polarization stimuli (LPS, 1 *μ*g/mL + IFN-*γ*, 50 ng/mL). Similar results were obtained in a second experiment, consisting of an analysis of pooled samples from three independent plates of BMDM, each one obtained from different animals. WT, wild type; KO, knockout; BMDM, bone marrow-derived macrophages; ELISA, enzyme-linked immunosorbent assay.

Arginase 1 to *β*-actin ratios were analyzed in protein extracts from both WT and KO-BMDM as a measure of macrophage activation. Basal levels of arginase 1 were higher in galectin-3 KO-BMDM (first lanes Fig. 5A, B and C). Upon IL-4 (50 ng/mL) or TGF*β*1 (50 ng/mL) stimulation, arginase 1 expression increased in WT-BMDM; whereas, this increase was not observed in galectin-3 KO-BMDM. It is interesting that exogenous galectin-3 (50 *μ*g/mL) did not restore this phenotype. WT-BMDM treated with IL-4 (50 ng/mL) and TGF*β*1 (50 ng/mL) increased Arg-I protein in these cells with a slightly additive effect of exogenous galectin-3 (50 *μ*g/mL) in response to IL-4 stimulus (Fig. 5A and B). In KO-BMDM, these same stimuli did not increase Arg-I protein levels. A large increase in Arg-I protein was detected in WT-BMDM and KO-BMDM stimulated with LPS 1*μ*g/mL plus IFN-*γ* 50 ng/mL with a little additive effect of exogenous galectin-3 (50 *μ*g/mL) just in WT-BMDM. Pro-M2 IL-4 (50 ng/mL) or M1 (LPS 1 *μ*g/mL plus IFN-*γ* 50 ng/mL) prototype signals did not increase VEGF protein secretion from both WT and KO -BMDM. After exposure to IL-4 (alternative activation of macrophages, M2), VEGF secretion was relatively more amplified in KO-BMDM than in WT-BMDM. Upon M1 activation, either WT-BMDM or KO-BMDM secreted the equivalent amounts of VEGF (Fig. 5D).

## Discussion

In this report, we have exploited a tumor model system developed by our own group consisting of a tumorigenic cell line Tm1, derived from a non-tumorignenic murine melanocyte cell line, melan-A 17,18. Among the many differences between melan-A and Tm1 cells 19, a striking difference was the loss of galectin-3 expression through hypermethylation of a CpG island composed of 33 CpG dinucleotides located at its 5′ upstream region in the melanoma cell. Moreover, some of these CpG dinucleotides are located within putative-binding sites to SP1 transcription factors, whose binding depends on CpG methylation 28. This very particular model system was generated by repeated cycles of adhesion/de-adhesion, which in turn led to epigenetic reprogramming 31. The DNA methylation status in the 5′ upstream region of galectin-3 gene was clearly associated with absence of mRNA and protein expression in Tm1 cells. Interestingly, DNA methylation encompassed all possible CpG dinucleotides present within the galectin-3 5′ upstream region. Others had shown that galectin-3 expression is controlled by DNA methylation 32, for example Ruebel et al. 33 showed that galectin-3 expression is epigenetically silenced by DNA hypermethylation in human pituitary tumors and Ahmed and Vasta showed it likewise in prostate cancer 32,34,35. Other members of the galectin family, such as galectin-1, can be silenced by DNA methylation and its re-expression induces apoptosis in cancer cells 36. These genes also exhibit a high density of CpG sites around their 5′ upstream region compatible with a role of DNA methylation in its transcriptional control. Here we showed that galectin-3 expression was lost in our model of melanoma progression. Although the precise mechanisms that target DNA methyltransferases (e.g., DNMT1) to a specific CpG island are still not clear, our results showed selective silencing of galectin-3 in murine melanoma. For some time, it was confusing in the literature, whether galectin-3 expression was increased or lost upon tumor progression. While there was a tendency to believe that galectin-3 would be lost in most epithelial tumors, a seminal work from Raz and coworkers 37 suggested that galectin-3 expression was not really lost in most carcinomas, but instead the epitope recognized by the most commonly used antibodies against galectin-3 was indeed processed by metalloproteases in the tumor microenvironment. Therefore, the apparent loss of galectin-3 was meant to be an artifact. Worthy of note is the fact that a recent paper from Brown and coworkers 38 studying human melanomas suggested that galectin-3 seems positively involved with melanoma progression to a large extent, confirming somehow data from Prieto and colleagues 39; however, in more advanced stages of melanomas, galectin-3 expression was lost 38. It is still not clear how galectin-3 expression is controlled in melanomas, obviously, it is possible that hypermethylation of its promoter may play a role in this process, though.

We next exploited the model system to further address what the selective advantage is of having tumor cells-expressing galectin-3 and if it is critical that the origin of galectin-3 is a tumor or a stromal cell. Our results demonstrated that melanoma cells expressing galectin-3 (Tm1G3) secreted larger amounts of VEGF in vitro than Tm1N3 cells, even without any specific stimulus. As far as we know, it is shown here for the first time that galectin-3 expression recovery in a melanoma cell increases VEGF secretion. Besides, BMDM from WT mice have secreted more VEGF than those derived from KO mice. Accordingly, WTG3 tumors are largest in volume, display larger functional vascular areas and have increased mRNA Arginase I levels to M2-stimulated macrophages. Noteworthy, when galectin-3-expressing tumor cells were engrafted in galectin-3 null mice, secretion of VEGF triggered in the tumor microenvironment was sufficient to provide for the necessary angiogenesis, allowing for the organization of a large functional vascular area and adequate response of arginase 1. Our in vivo results extend what Markowska et al. 4 showed in vitro that galectin-3 siRNA knockdown as well as galectin-3 blockers resulted in reduction in angiogenesis induced by VEGF and bFGF mediators. In this regard, galectin-3 from tumor cells could orchestrate cellular and tissue events, including recruitment of monocytes to the tumor microenvironment. VEGF, from both tumor cells and monocyte/macrophage origin would then be a key mediator of angiogenesis and maintenance of an immature status of the immune system within the tumor, thus favoring tumor growth. The main response to VEGF in vivo is mediated by VEGF receptor-2 (VEGFR2) and the way it regulates angiogenesis is through VEGFR2 expression and its activation in cells. We observed that VEGFR2 and its phosphorylated form were increased just in WTG3 and KOG3 tumors suggesting that galectin-3 from tumor cells had a crucial impact in VEGF/VEGFR2 cell. Altogether these results point that although galectin-3 from both tumor parenchyma and tumor stroma may support tumor growth, as it would be expected for a secreted protein, acting in the extracellular *milieu*, galectin-3 from parenchymal (tumor) cells may be of greater impact to tumor-associated angiogenesis. Despite, its potential role within tumor cells, it is interesting to note that galectin-3 of tumor origin may modify signaling from stromal cells. Markowska et al. 5 hypothesized that galectin-3 organize VEGFR2-clusters that potentiate cell signaling effects in endothelial cells. Our results essentially extend the findings of the Panjwani group in a melanoma model 3,4. Of interest, a very recent paper from the Salmon group demonstrated that galectin-3 interferes with both VEGFR1 and VEGFR2 signaling in endothelial cells 40. These results strengthen the notion that galectin-3 interactions are potential targets for intervention in tumors.

While in galectin-3-expressing microenvironments, it was possible to observe CD68-positive cells infiltrating the tumor mass, these cells were only found in the periphery of galectin-3 negative tumors engrafted in KO mice. Therefore, the presence of galectin-3 interferes the pattern of recruitment and topography of cells infiltrating tumors, such as monocytes and macrophages. In a recent paper 41, a very similar finding was described and a critical issue then would be the polarity of differentiation of macrophages within the tumor microenvironment. In our experiments, BMDM from both WT and KO behave differently before TGF*β*1 stimulus, regarding VEGF expression, following the same tendency observed in in vivo experiments. Interestingly, in the absence of galectin-3, TGF*β*1 signaling seems altered in several pathophysiological contexts, as illustrated by the poor fibrogenic response in the liver of chronically infected mice, for example 42,43. WT macrophages responded to TGF*β*1 increase with increased VEGF secretion and galectin-3 expression, confirming that WT macrophages receive these TGF*β*1 signals. Besides Tm1N3 cells are less responsive to TGF*β*1 with modest increase in VEGF secretion upon TGF*β*1 stimulus. Gong et al. 44 published recently that TGF*β*1-receptor-I KO mice have decreased galectin-3 levels after M2-prone stimuli arguing in favor that there might exist some putative role of galectin-3 in the regulation of TGF*β*1-dependent pathways.

**Figure 6:**
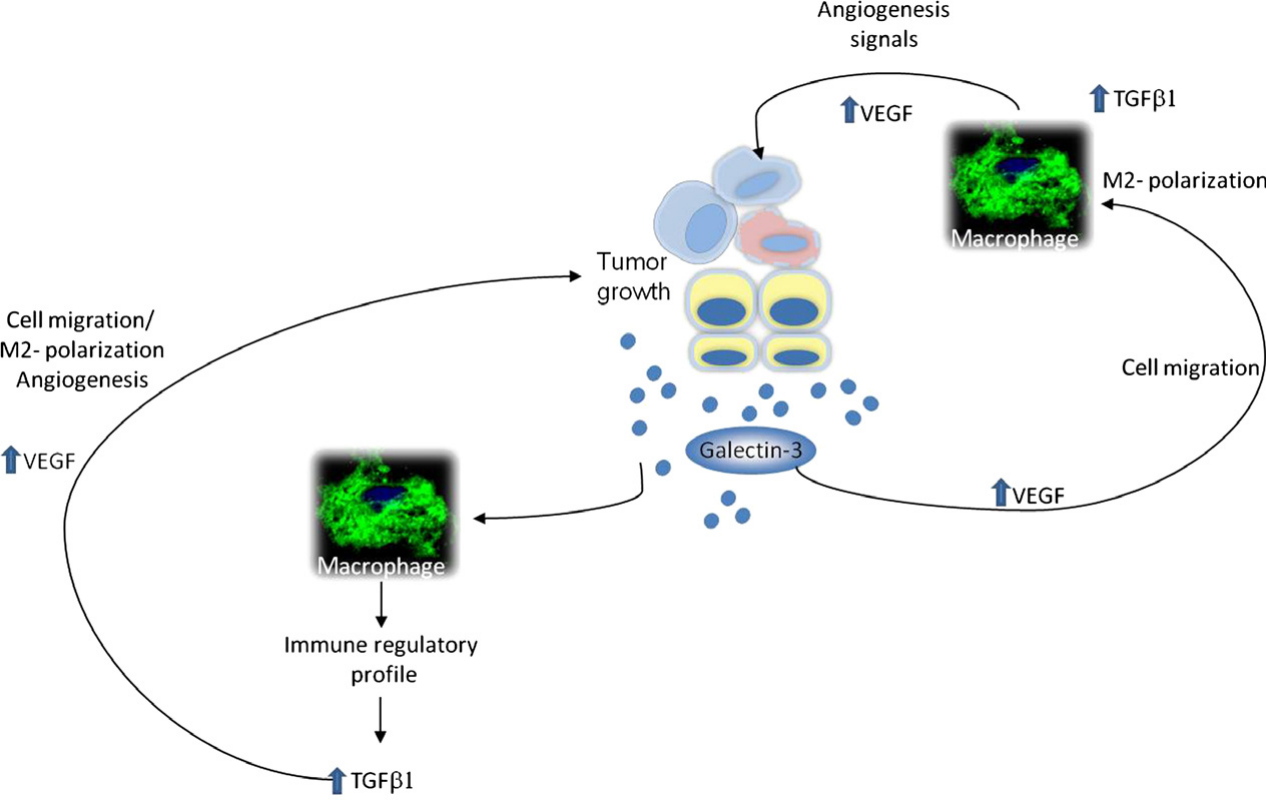
Diagram representing our hypothesis that galectin-3 from tumor parenchyma or stroma in the tumor microenvironments sites could augment VEGF signals to: improve angiogenesis, enhance macrophage migration to tumors, which in turns enhance TGF*β*1 signaling to produce more VEGF. In addition, galectin-3 could enhance macrophages to secreted cytokines to an immune regulatory pattern with TGF*β*1 as a mediator, which in turns enhances VEGF and so on.

Finally, we have evaluated in vitro aspects of the polarity of the macrophage response to known stimuli, which are processed toward either an M1 or an M2 response in both WT and KO-BMDM. Curiously, basal level accumulation of arginase 1 in KO-BMDM was higher than in WT-BMDM. Upon activation with M2 stimuli, WT-BMDM processed the signals and accumulated arginase 1, whereas in the absence of galectin-3, arginase 1 protein expression was decreased. It is likely that this effect was due to galectin-3 functioning intracellularly, as exogenously added recombinant galectin-3 did not interfere with the result on KO-BMDM. Note that an apparent discrepancy exists as we compare the global levels of arginase expression in vivo, as performed in Figure 4, and the production of arginase, as evaluated by Western blots in protein extracts of macrophage differentiated and activated in vitro, as shown in Figure 5. Data in Figure 4 represent different subpopulations of macrophages present within all the distinct tissue subcompartments of tumors. Dissection of the different tissue contexts within a tumor and detailed analysis of the macrophage phenotype within each context is warranted. Our results, as summarized in Figure 6, support the notion that galectin-3 is part of the alternative activation pathway of macrophages (M2 phenotype), as in its absence this phenotype is dysregulated. In conclusion, regardless its source (tumor parenchyma or stroma), galectin-3 plays a role in the organization of the tumor microenvironment. Decreased expression of galectin-3 in either compartment may lead to impaired tumor angiogenesis, as we have observed experimentally, as a result of diminished VEGF and/or resistance to TGF*β*1. More studies related to these phenomena are necessary to elucidate what is the exact point that this galectin-3 disruption could benefit patients, thereby providing a window for improved chemotherapy treatment.

## Supporting Information

Figure S1 (A) Methylation status of region shown in A after genomic sequencing of bisulfite-treated DNA from Melan-A and Tm1 cells. (B) Overexpression of galectin-3 melan-A in parental lineage and down expression in Tm1. Tm1 cell line treated with increased concentrations of the demethylanting agent 5′-Aza-2-deoxycytidine (5′- Aza-dCR) for 72 h showing that galectin-3 expression was only restored with the largest concentration of 5′-Aza- dCR in PCR. (D) Analysis. Decreased methylation in galectin-3 promoter sequence after treatment of Tm1 cells with 10 μmol/L of 5′-Aza-dCR. Tm1 cells were stably transfected with 1 μg of human galectin-3 gene cloned in pEF1-neo/gal-3 (G3) or pEF1-neo (N3) in RPMI containing 5% of fetal bovine serum and geneticin, G418 (Sigma) 1 mg/mL. (F) The western blotting assay shows that galectin-3 were expressed in Melan-A cells as well as in Tm1G3 or pEF1-neo/gal-3 (G3) cells.

Figure S2 All DEG's can be seen in Heatmaps where each square represent one independent animal where red indicates increase and blue indicates decrease. The differentially expressed genes (DEG's) in the five comparisons (listed in the table) showing no differences in gene expression profile between WT and KO animals. The comparisons: WTG3 versus WTN3 and KOG3 versus KON3 showed differentially expressed transcripts and the transcripts identified as differentially expressed were determined with adjusted *P <* 0.1 and fold change >1.3. The Venn diagram shows that 11 transcripts (intersection) sustain similar DEG's in both models. Independent DEG's were observed in just WTG3 versus WTN3 (33) or KOG3 versus KON3 (9) as represented in the diagram.

Figure S3 Evaluation of VEGFR2 and phosphorilated- VEGFR2 (PY1214) which as pro-angiogenic receptor of VEGF mediators. The expression was detected by immunoblotting from total tumors protein extraction (20 μg/lane). Each lane represented one representative result from a number of animals/group (*n* were represented inside the legend). Densitometric analysis was performed using ImageJ and the results in each graph corresponded to mean ± SEM and the results were analyzed by t unpaired test, two-tailed with **P* < 0.1; ***P* < 0.01 and ****P* < 0.001.

Figure S4 (A) Western blotting detection of galectin-3 of WT-BMDM and (B) Tm1N3 mock-cells or Tm1G3 galectin-3 transfected cells of total protein cells extractions (50 μg/lane) after TGFβ1 stimuli (0, 25, 50, 100 ng/mL). These results were representative of two independent experiments. The number above each lane represents the galectin-3/β-actin ratio from densitometry analysis were performed using ImageJ.

Figure S5 No significant differences in IL4, IL10, IL12p40, and INFγ mRNA. These results were expression levels from three distinct individuals. The graphs corresponded to mean ± SEM from three distinct animals in each group and the results were analyzed by *t* unpaired test.

Figure S6 Immunofluorescence for CD68+ cells (in green) in WTG3, WTN3, KOG3 and KON3 tumors. Note CD68+ inner area macrophages (above) and peripherical macrophages (bellow) tumor-associated cells. The graphs corresponded to mean ± SEM from three distinct animals in each group and the results were analyzed by t unpaired test.

Table S1 List of primers for semi-quantitative PCR and qPCR.
