# Supplementary figures and images for: Galectin-3 disruption impaired tumoral angiogenesis by reducing VEGF secretion from TGFβ1-induced macrophages
Source: Cancer Med. 2014 Jan 12;3(2):201–14. doi: 10.1002/cam4.173 (PMC3987071; doi:10.1002/cam4.173)

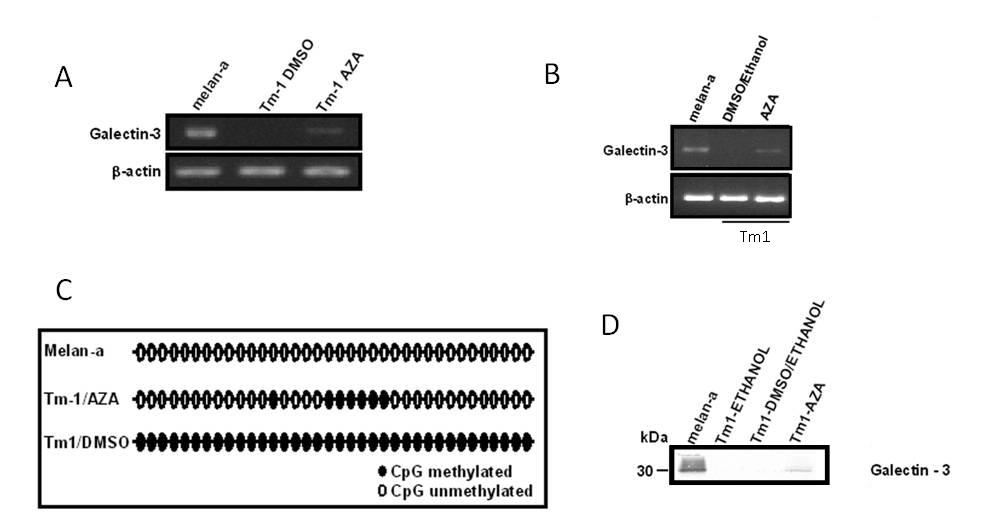

Supplement: Figure S1 — (A) Methylation status of region shown in A after genomic sequencing of bisulfite-treated DNA from Melan-A and Tm1 cells. (B) Overexpression of galectin-3 melan-A in parental lineage and down expression in Tm1. Tm1 cell line treated with increased concentrations of the demethylanting agent 5′-Aza-2-deoxycytidine (5′- Aza-dCR) for 72 h showing that galectin-3 expression was only restored with the largest concentration of 5′-Aza- dCR in PCR. (D) Analysis. Decreased methylation in galectin-3 promoter sequence after treatment of Tm1 cells with 10 μmol/L of 5′-Aza-dCR. Tm1 cells were stably transfected with 1 μg of human galectin-3 gene cloned in pEF1-neo/gal-3 (G3) or pEF1-neo (N3) in RPMI containing 5% of fetal bovine serum and geneticin, G418 (Sigma) 1 mg/mL. (F) The western blotting assay shows that galectin-3 were expressed in Melan-A cells as well as in Tm1G3 or pEF1-neo/gal-3 (G3) cells. [file cam40003-0201-sd1.jpg]

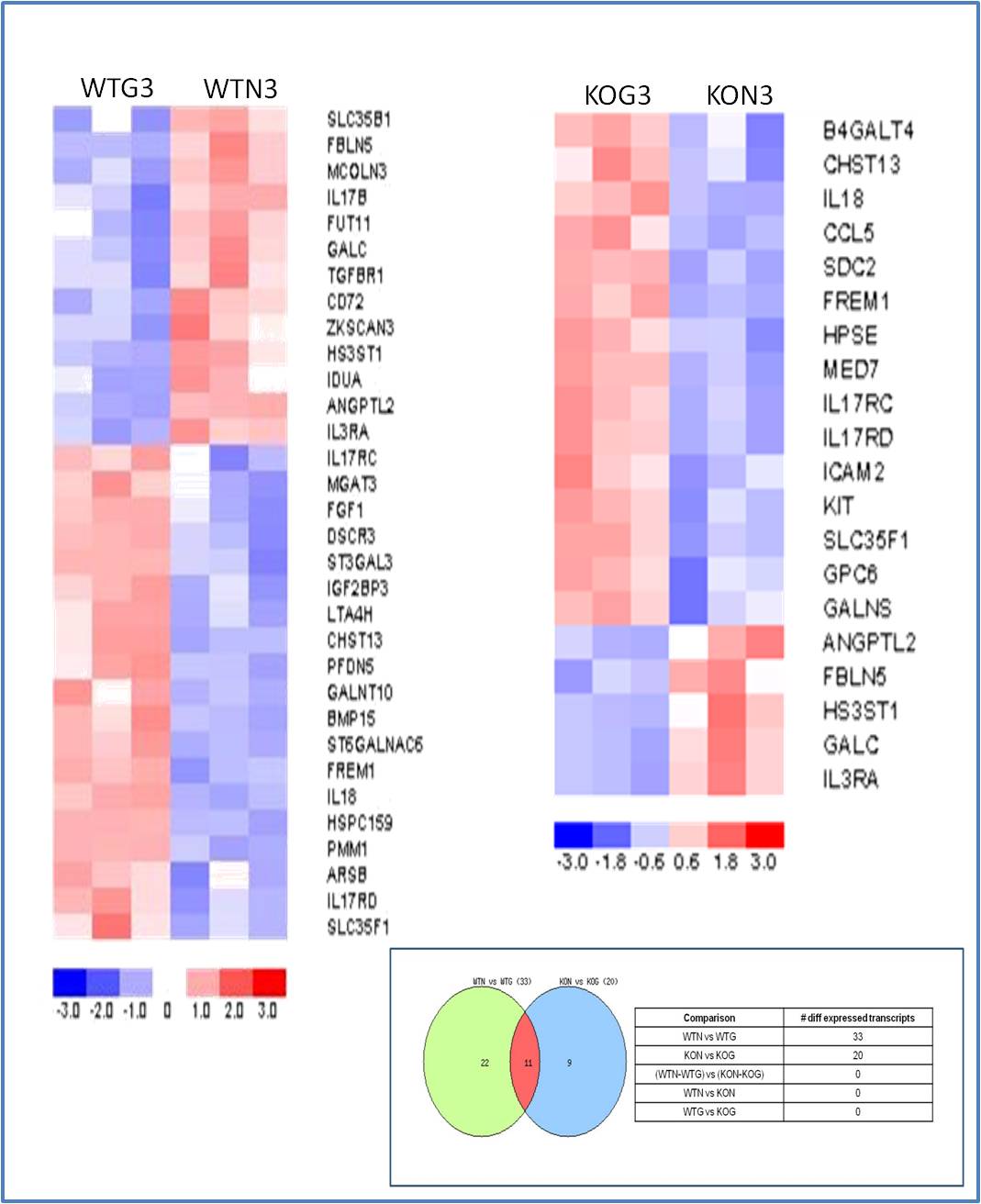

Supplement: Figure S2 — All DEG's can be seen in Heatmaps where each square represent one independent animal where red indicates increase and blue indicates decrease. The differentially expressed genes (DEG's) in the five comparisons (listed in the table) showing no differences in gene expression profile between WT and KO animals. The comparisons: WTG3 versus WTN3 and KOG3 versus KON3 showed differentially expressed transcripts and the transcripts identified as differentially expressed were determined with adjusted P < 0.1 and fold change >1.3. The Venn diagram shows that 11 transcripts (intersection) sustain similar DEG's in both models. Independent DEG's were observed in just WTG3 versus WTN3 (33) or KOG3 versus KON3 (9) as represented in the diagram. [file cam40003-0201-sd2.jpg]

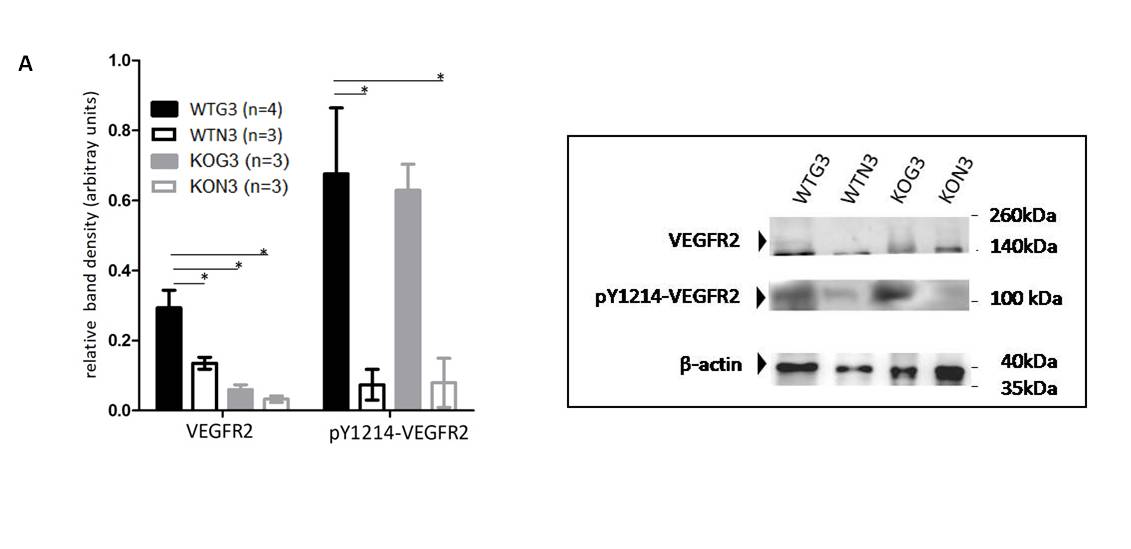

Supplement: Figure S3 — Evaluation of VEGFR2 and phosphorilated- VEGFR2 (PY1214) which as pro-angiogenic receptor of VEGF mediators. The expression was detected by immunoblotting from total tumors protein extraction (20 μg/lane). Each lane represented one representative result from a number of animals/group (n were represented inside the legend). Densitometric analysis was performed using ImageJ and the results in each graph corresponded to mean ± SEM and the results were analyzed by t unpaired test, two-tailed with *P < 0.1; **P < 0.01 and ***P < 0.001. [file cam40003-0201-sd3.jpg]

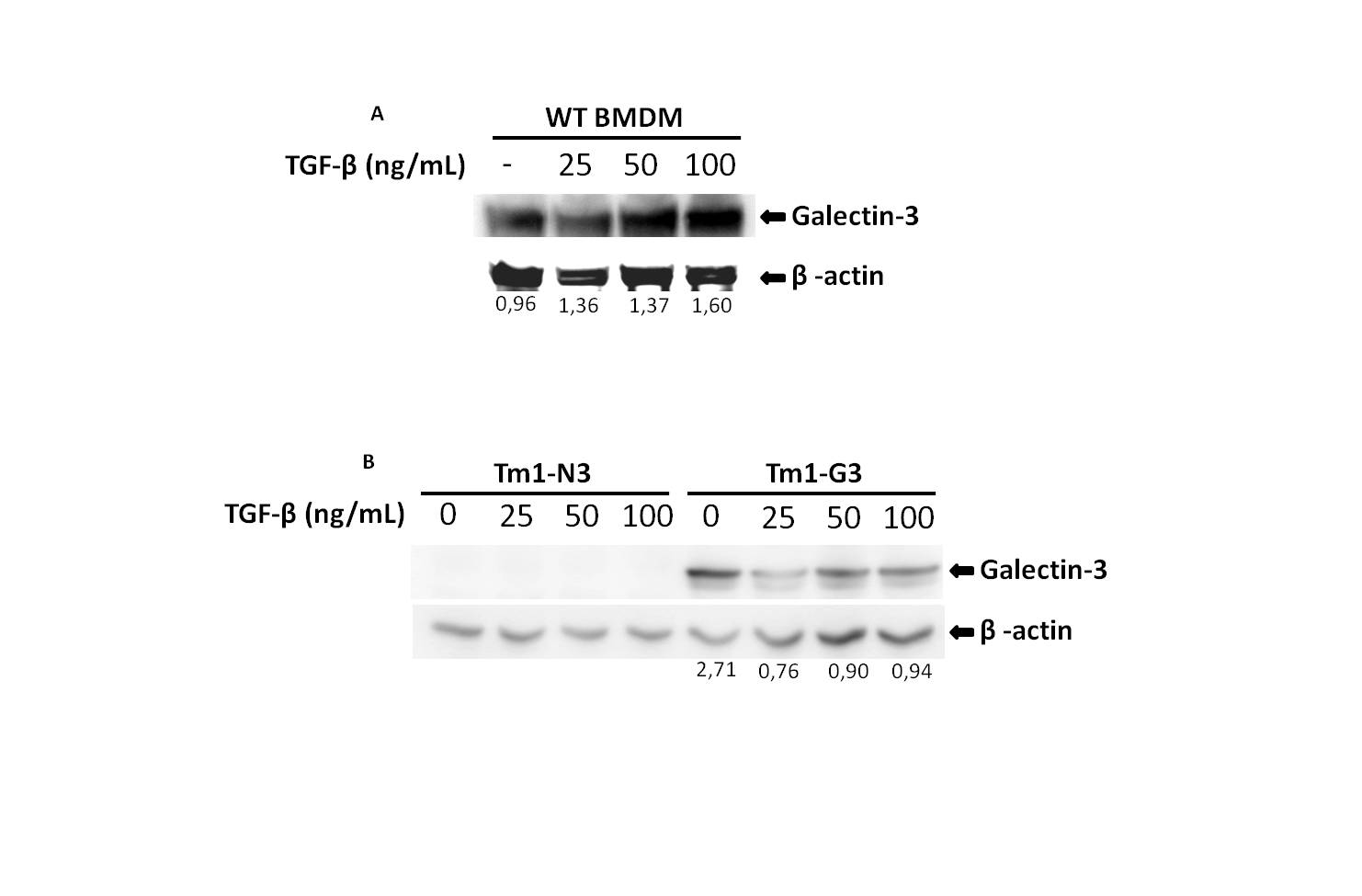

Supplement: Figure S4 — (A) Western blotting detection of galectin-3 of WT-BMDM and (B) Tm1N3 mock-cells or Tm1G3 galectin-3 transfected cells of total protein cells extractions (50 μg/lane) after TGFβ1 stimuli (0, 25, 50, 100 ng/mL). These results were representative of two independent experiments. The number above each lane represents the galectin-3/β-actin ratio from densitometry analysis were performed using ImageJ. [file cam40003-0201-sd4.jpg]

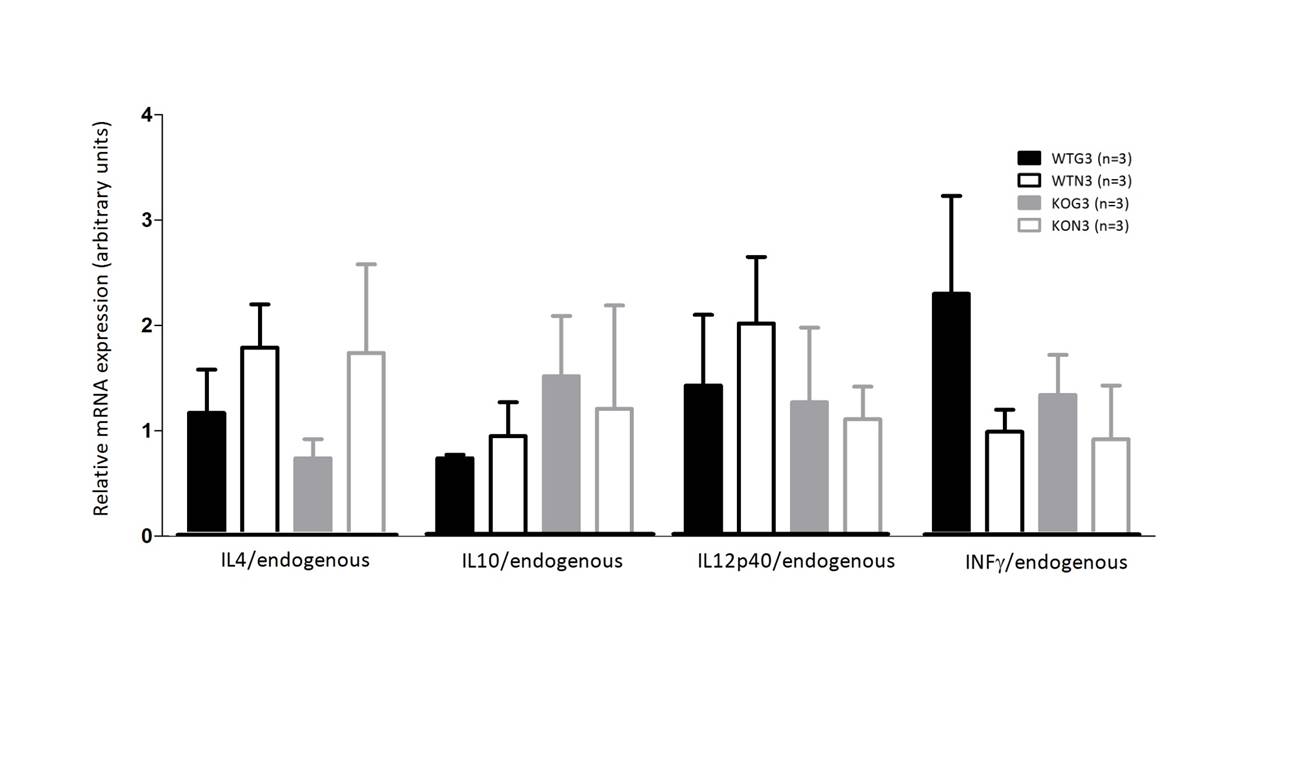

Supplement: Figure S5 — No significant differences in IL4, IL10, IL12p40, and INFγ mRNA. These results were expression levels from three distinct individuals. The graphs corresponded to mean ± SEM from three distinct animals in each group and the results were analyzed by t unpaired test. [file cam40003-0201-sd5.jpg]

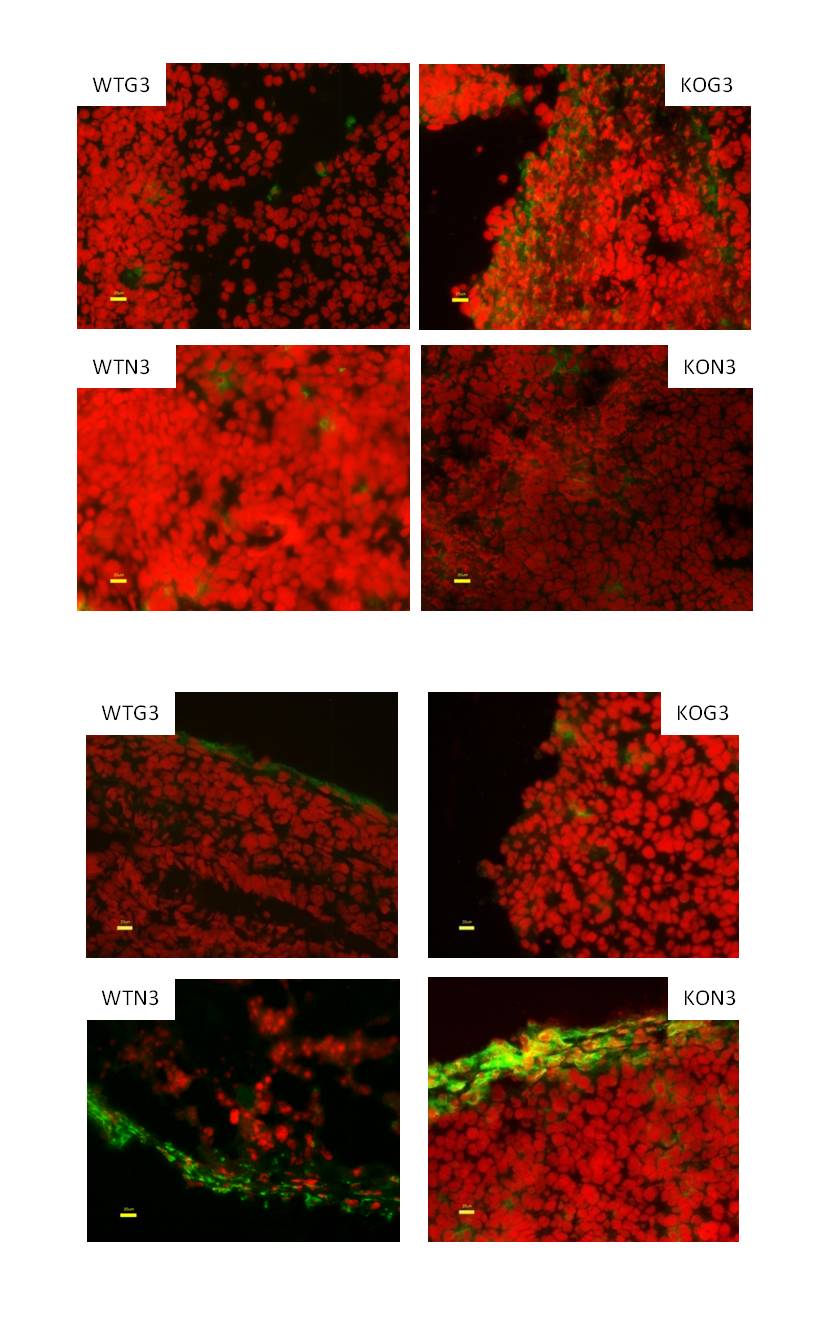

Supplement: Figure S6 — Immunofluorescence for CD68+ cells (in green) in WTG3, WTN3, KOG3 and KON3 tumors. Note CD68+ inner area macrophages (above) and peripherical macrophages (bellow) tumor-associated cells. The graphs corresponded to mean ± SEM from three distinct animals in each group and the results were analyzed by t unpaired test. [file cam40003-0201-sd6.jpg]

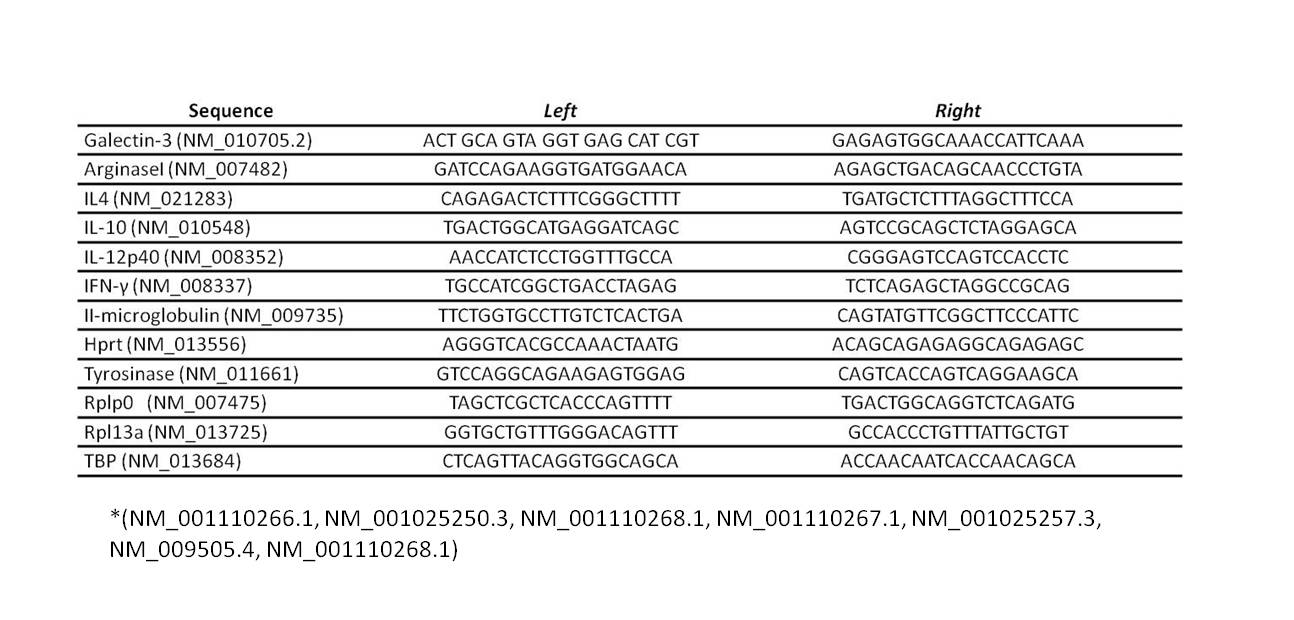

Supplement: Table S1 — List of primers for semi-quantitative PCR and qPCR. [file cam40003-0201-sd7.jpg]
