# Supplementary material for: Galectin-3 disruption impaired tumoral angiogenesis by reducing VEGF secretion from TGFβ1-induced macrophages
Source: Cancer Med. 2014 Jan 12;3(2):201–14. doi: 10.1002/cam4.173 (PMC3987071; doi:10.1002/cam4.173)
Supplement: Data S1 — Supplementary materials and methods. [file cam40003-0201-sd8.docx]

**Supporting Information**

**Supplementary materials and methods**

**Galectin-3 DNA methylation analysis**

In order to find putative CpG islands in murine and human galectin-3 gene promoter in Melan-a and Tm1 cells the CpG plot software (htpp://www.ebi.ac.uk/emboss/cpgplot) for *in silico* analyses was used. The parameters used were: (1) C/G ratio > 0.6; (2) C+G frequency > 50%; (3) sequence length > 100 nucleotides. Sp1 binding sites were also identified *in silico* using the AliBaba 2.1 software (htpp://www.gene-regulation.com).

Genomic DNA from both Melan-a and Tm1 cells were obtained by digestion with proteinase K (100 μg/mL - Life Technologies,CA) and RNAse (20μg/Ml- Life Technologies,CA) for 16 hours at 50^o^C, followed by phenol/chloroform/isoamyl alcohol extraction. The presence of CpG islands in galectin-3 promoter sequence, was detected by the bisulfite genome sequencing method. Briefly, 1μg of DNA was treated with sodium bisulfite (2.5 M sodium bisulfate, 1M hydroquinone, 2 M NaOH) for 3 hours at 70^o^C. DNA was purified using a silica-based membrane (Wizard minicolumns, Promega) according to manufacturer’s instructions. Nested-PCR was performed using 2 sets of primers for galectin-3 (Table 1) in a 25μL final volume containing 1X PCR buffer (Invitrogen, Life Technologies, Carlsbad, CA), 1.5 mM MgCl_2_ (Invitrogen, Life Technologies, Carlsbad, CA), 200 µM dNTPs (Invitrogen, Life Technologies, Carlsbad, CA), 0.32 μM of each primer and 1U of Taq platinum (Invitrogen, Life Technologies, Carlsbad, CA). The PCR conditions were: 94^o^C for 12 min, 1 cycle at 94^o^C for 3 min, 50^o^C for 3 min, 72^o^C for 2 min, 5 cycles at 94^o^C for 3 min, 52^o^C for 3 min, 72^o^C for 2 min and 35 cycles at 94^o^C for 1 min, 55^o^C for 1 min, 72^o^C for 1 min and 72^o^C for 6 min. One μl of the first reaction was used for the second PCR reaction which was performed under the following conditions: 94^o^C for 12 min, 1 cycle at 94^o^C for 3 min, 53^o^C for 3 min, 72^o^C for 2 min, 5 cycles at 94^o^C for 3 min, 55^o^C for 3 min, 72^o^C for 2 min, 35 cycles at 94^o^C for 1 min, 58^o^C for 1 min, 72^o^C for 1 min and 72^o^C for 6 min. Amplicons were purified using the QIAquick Gel Extraction kit (Qiagen) and cloned in a T vector (pGEM-Teasy Vector, Promega). Twelve positive clones were selected and sequenced using the Dynamic ^TM^ ET terminator cycle sequencing kit (Amersham/GE Healthcare) and the ABI 300 sequencer (Applied Biosystems, Life Technologies, Carlsbad, CA). Tm1 cells were cultured with 5´-aza-2´-deoxycytidine (2.5; 5 or 10 μM; Sigma-Aldrich, St. Louis, MO,USA) for 72 hours. For the treatment with trichostatin A (Sigma-Aldrich, St. Louis, MO,USA), Tm1 cells were pre-treated with 10μM 5´-aza-2´-deoxycytidine for 48 hours when different concentrations of trichostatin A (50, 100 and 200nM) or 4-phenylbutyrate acid (1, 2, 3 and 4mM, Merck Millipore, United Kingdom) were added to the media for 24 hours. Alternatively, Tm1 cells were treated only with 4-phenylbutyrate acid (1, 2, 3 and 4mMm, Merck Millipore, United Kingdom) for 72 hours.

**Glycoarray study**

Tumors from at least three individuals were sectioned into four quadrants and total RNA was extracted from tissue sections weighing 40 - 100mg using Tryzol reagent (Invitrogen Life Technologies, Carlsbad, CA). The RNA was purified with RNeasy mini-kit (Qiagen, Austin, TX, USA) according to the manufacturer’s protocol. RNA integrity was verified on a 1% agarose gel and ribosomal bands were visualized by ethidium bromide staining under UV light. RNA quantification was measured using a spectophotometer. After extraction and cleanup, mRNAs were sent to Consortium for functional Glycomics where the microarray reaction was performed (http://www.functionalglycomics.org/static/consortium/consortium.shtml). The GlycoV4 array focused array includes probes for ~1200 mouse probe-ids related to glyco-genes. This array does not contain mismatched probes. Data normalization was performed using RMA Express 1.0 with quantile normalization, median polish and background adjustment. The Limma package [1] in the R software was used to find transcripts with differential expression. Fold changes and standard errors were estimated by fitting a linear model for each gene and empirical Bayes smoothing was applied to the standard errors. Results are presented between two or more experimental conditions as fold change in expression level, the moderated t-statistic, the *p-*value, and the adjusted *p-*value. The adjusted p-value is the p-value adjusted for multiple testing using the Benjamini and Hochberg’s [2] method to control the false discovery rate of 0.1 or less. Heatmaps were generated with dChip program (www.dChip.org). All data from microchip reading are available in the Glycomics site (<http://www.functionalglycomics.org/glycomics/publicdata/microarray.jsp>).

**Specimens’ preparation, sections and Hematoxylin and Eosin (H/E) stain**

After tumor growth evaluation, tumors were surgically removed and specimens were fixed (3.7% formaldehyde), embedded in 70% ethanol followed by dehydration and inclusion in paraffin. Tumors were sectioned (thickness 3-5 μm) with a microtome (Leica, Leica Microsystems, Wetzlar, Germany) and collected onto treated glass slides and stored at Room Temperature for further analysis or H/E (Haematoxylin /Eosin) like as described elsewhere.

**Immunohistochemistry**

Glass slides were rinsed three times in phosphate buffer saline (PBS), pH 7.4 at RT, and treated with 3% H_2_O_2_ in methanol to suppress endogenous peroxidase activity. After being washed in PBS, slides were next treated with 1% BSA at RT for 1 hour. The slides were washed again in PBS, and then incubated overnight at 4^o^C with anti-mouse CD34 (rat anti-mouse monoclonal antibody, GeneTex, Irvine, CA). After that, the slides were washed with PBS and incubated with SuperPicture™ Kit (Invitrogen, Life Technologies, Carlsbad, CA) – a biopolymer with reactivity to rat, mouse and rabbit primary antibodies labeled with horseradish peroxidase) for 1 hour at 37^o^C. All these incubations were carried out in a humidified chamber. Peroxidase enzyme activity was developed by DAB (3,3-diaminobenzidine -substrate-chromogen solution - Sigma-Aldrich, St. Louis, MO,USA).Distilled water was added to stop peroxidase reaction. The slides were then weakly counter-stained with Harris’ Haematoxylin, dehydrated in an ethanol series and mounted in Permount™ Mounting Medium (e-bioscience, San Diego, CA, USA). For each batch, one positive and negative controls (suppression of primary antibody) was included to validate these results.

**Immunofluorescence**

After tumors excisions, specimens were embebed in Tissue Tek® (Leica, Leica Microsystems, Wetzlar, Germany) and stored at -80^o^C. Then they were sectioned (5-7 μm thickness) with a cryostat (Leica, Leica Microsystems, Wetzlar, Germany) and collected onto treated glass slides and maintained at -80^o^C. Slides were thawed, fixed in cold ethanol, rehydrated in PBS 7.4 pH, and incubated with 2.5% BSA in PBS. After three washes with PBS, the slices were incubated with a rat anti-CD68 (a murine monocytes/tissue macrophages marker) (AbCam,Calsburg,USA) (1:150) primary antibody for 1h at 37 ^o^C in a humidity chamber. After three washes in PBS, the slices were incubated with a secondary fluorescent-Alexa-588 goat-anti-rat antibody (Invitrogen, Life Technologies, Carlsbad, CA) for 2h, followed by DAPI incubation. For each batch, one positive and negative control (suppression of primary antibody) was included to validate these results.

**Western Blotting**

For total protein extraction, tumor samples were homogenized in 1% Triton X-100, 1% sodium deoxicolate, 150mM NaCl, 1% SDS, 10mM NaF, 50mM Tris-HCl, 2μg/ml aprotinin, 1mM PMSF (phenylmethanesulfonylfluoride) (Sigma-Aldrich, St. Louis, MO,USA) and 1mM sodium ortovanadate (Sigma-Aldrich, St. Louis, MO,USA). Insoluble materials were removed by 13000g centrifugation at 4^o^C for 15 minutes. Protein concentration was determined using biscinchoninic acid (Thermo Fisher Scientific Inc, New York, USA) method. Total protein extracts from each sample (20ug) were eletrophoretically separated in SDS-PAGE and blotted onto PVDF (Hybond-P, GE Healthcare, Little Chalfont, United Kingdom), membrane according to standard procedures. Membranes were blocked with PBS- 0.1% Tween®20 (Sigma-Aldrich, St. Louis, MO,USA) containing 5% BSA and then incubated with antibodies against VEGF (1:500; Santa Cruz biotechnology, CA), VEGFR2 and against its phosphorylated form, pY1214-VEGFR2 (1:500; Invitrogen, Life Technologies, Carlsbad, CA), TGFβ1 (1:3000; BD Biosciences- Pharmingen in San Diego,CA) or Arginase-I (1:1000; BD Biosciences- Pharmingen in San Diego, CA) in PBS- 0.1% Tween®20 (Sigma-Aldrich, St. Louis, MO,USA)-1% BSA overnight at 4ºC. Membranes were washed with PBS- 0.1% Tween®20 (Sigma-Aldrich, St. Louis, MO,USA) and incubated with specific HRP (horseradish peroxidase) labeled secondary antibody (Sigma-Aldrich, 1:4000). Reactive bands were detected with luminol and H_2_O_2_ reagents and visualized using an image capture system (Image Quant LAS 4000, GE Health care).

**Images acquisition and Quantification**

All images were captured with an Olympus microscope connected to an image Nikon DMX1200 acquiring system using specific software (ACT-1 software®). From all samples, 20 independent fields were acquired in high resolution where each pixel corresponded to 0.32µm according to ACT-1 software® manufacturer’s instructions. Total field area was equal to 4.2×10^5^ µm^2^, equivalent to 80 crosses grid in ImageJ software 1.44d (National Institute of Health, USA). The necrosis area quantification was determined as previously described [3]. Functional vessels area was estimated from the median calculated for the total number of crosses counted inside CD34-positive vascular structures with erythrocytes or cells inside then in each sample multiplied by total field area described above and then divided by 80. All data were statistically analyzed as described below.

**Supplementary Figure Legends**

Supporting information Fig. S1 (A) Methylation status of region shown in A after genomic sequencing of bisulfite treated DNA from Melan-a and Tm1 cells. (B) Overexpression of galectin-3 melan-a in parental lineage and down expression in Tm1. Tm1 cell line treated with increased concentrations of the demethylanting agent 5’-Aza-2-deoxycytidine (5'-Aza-dCR) for 72 hours showing that galectin-3 expression was only restored with the largest concentration of 5'-Aza-dCR in PCR. (D) analysis. Decreased methylation in galectin-3 promoter sequence after treatment of Tm1 cells with 10 μM of 5'-Aza-dCR. Tm1 cells were stably transfected with 1µg of human galectin-3 gene cloned in pEF1-neo/gal-3 (G3) or pEF1-neo (N3) in RPMI containing 5% of fetal bovine serum and geneticin, G418 (Sigma) 1mg/mL. (F) The western blotting assay shows that galectin-3 were expressed in Melan-a cells as well as in Tm1G3 or pEF1-neo/gal-3 (G3) cells.

Supporting information Fig. S2: All DEG´s can be seen in Heatmaps where each square represent one independent animal where red indicates increase and blue indicates decrease. The differentially expressed genes (DEG's) in the five comparisons (listed in the table) showing no differences in gene expression profile between WT and KO animals. The comparisons: WTG3 vs. WTN3 and KOG3 vs KON3 showed differentially expressed transcripts and the transcripts identified as differentially expressed were determined with adjusted p-value < 0.1 and fold change > 1.3. The Venn diagram shows that 11 transcripts (intersection) sustain similar DEG´s in both models. Independent DEG´s were observed in just WTG3 versus WTN3 (33) or KOG3 versus KON3 (9) as represented in the diagram.

Supporting information Fig. S3: Evaluation of VEGFR2 and phosphorilated-VEGFR2 (PY1214) which as pro-angiogenic receptor of VEGF mediators. The expression was detected by immunoblotting from total tumors protein extraction (20µg/lane). Each lane represented one representative result from a number of animals/group (n were represented inside the legend). Densitometric analysis was performed using ImageJ and the results in each graph corresponded to mean ±SEM and the results were analyzed by t unpaired test, two-tailed with *p<0,1;**p,0,01 and ***p<0,001.

Supporting information Fig. S4: (A) Western blotting detection of galectin-3 of WT-BMDM and (B) Tm1N3 mock-cells or Tm1G3 galectin-3 transfected cells of total protein cells extractions (50μg/lane) after TGFβ1 stimuli (0ng/mL, 25ng/mL,50ng/mL, 100ng/mL). These results were representative of two independent experiments. The number above each lane represents the galectin-3/β-actin ratio from densitometry analysis were performed using ImageJ.

Supporting information Fig. S5: No significant differences of IL4, IL10, IL12p40 and INFγ mRNA. These results were expression levels from three distinct individuals. The graphs corresponded to mean ±SEM from three distinct animals in each group and the results were analyzed by t unpaired test.

Supporting information Fig. S6: Immunofluorescence for CD68+ cells (in green) in WTG3, WTN3, KOG3 and KON3 tumors. Note CD68+ inner area macrophages (above) and peripherical macrophages (bellow) tumor associated cells. The graphs corresponded to mean ±SEM from three distinct animals in each group and the results were analyzed by t unpaired test.

Table S1: **List of primers for semi-quantitative PCR and qPCR**

**References**

1. Smyth GK. Linear models and empirical bayes methods for assessing differential expression in microarray experiments. Stat Appl Genet Mol Biol. 2004; Feb 12 (3).
2. Benjamini Y. and Hochberg Y. Controlling the false discovery rate: a practical and powerful approach to multiple testing. J. R. Statist. Soc. B, 1995; 57:289–300.
3. Otake AH, Mattar AL, Freitas HC, Machado CM, Nonogaki S, Fujihara CK, Zatz R, Chammas R. Inhibition of angiotensin II receptor 1 limits tumor-associated angiogenesis and attenuates growth of murine melanoma. Cancer Chemother Pharmacol. 2010 May;66(1):79-87.
